# Diversity of Microbial Functional Genes Promotes Soil Nitrogen Mineralization in Boreal Forests

**DOI:** 10.3390/microorganisms12081577

**Published:** 2024-08-02

**Authors:** Xiumin Zhang, Huayong Zhang, Zhongyu Wang, Yonglan Tian, Wang Tian, Zhao Liu

**Affiliations:** 1 Research Center for Engineering Ecology and Nonlinear Science, North China Electric Power University, Beijing 102206, China; 120192132233@ncepu.edu.cn (X.Z.); zhy_wang@ncepu.edu.cn (Z.W.); yonglantian@ncepu.edu.cn (Y.T.); tianwang@ncepu.edu.cn (W.T.); 2 Theoretical Ecology and Engineering Ecology Research Group, School of Life Sciences, Shandong University, Qingdao 266237, China; liuzhao9555@sdu.edu.cn

**Keywords:** functional genes, microbial community diversity, nitrogen transformations, soil nitrogen mineralization, soil properties

## Abstract

Soil nitrogen (N) mineralization typically governs the availability and movement of soil N. Understanding how factors, especially functional genes, affect N transformations is essential for the protection and restoration of forest ecosystems. To uncover the underlying mechanisms driving soil N mineralization, this study investigated the effects of edaphic environments, substrates, and soil microbial assemblages on net soil N mineralization in boreal forests. Field studies were conducted in five representative forests: *Larix principis-rupprechtii* forest (LF), *Betula platyphylla* forest (BF), mixed forest of *Larix principis-rupprechtii* and *Betula platyphylla* (MF), *Picea asperata* forest (SF), and *Pinus sylvestris* var. *mongolica* forest (MPF). Results showed that soil N mineralization rates (R_min_) differed significantly among forests, with the highest rate in BF (*p* < 0.05). Soil properties and microbial assemblages accounted for over 50% of the variability in N mineralization. This study indicated that soil environmental factors influenced N mineralization through their regulatory impact on microbial assemblages. Compared with microbial community assemblages (α-diversity, Shannon and Richness), functional genes assemblages were the most important indexes to regulate N mineralization. It was thus determined that microbial functional genes controlled N mineralization in boreal forests. This study clarified the mechanisms of N mineralization and provided a mechanistic understanding to enhance biogeochemical models for forecasting soil N availability, alongside aiding species diversity conservation and fragile ecosystem revitalization in boreal forests.

## 1. Introduction

Soil nitrogen (N) mineralization, the process of microbial transformation from organic N to inorganic N (NH_4_^+^-N and NO_3_^−^), is the key process that influences ecosystem functions (such as forest productivity and soil fertility) and biogeochemistry of N in ecosystems [1,2,3,4,5,6,7]. Soil net nitrogen mineralization is estimated to account for 47% to 68% of the total N requirement in forest ecosystems in terrestrial biomes [8], which is widely considered as the key process of soil N cycling in forest ecosystems [9].

Soil N mineralization is regulated by various factors, such as soil properties and microbial properties [10,11,12,13]. Soil N mineralization rate (R_min_) is considered to be mediated by environmental factors through altering the traits of microbial communities [14,15,16,17]. On one hand, environmental factors can directly alter R_min_. For example, soil N mineralization is generally suppressed by soil acidification [18] while the addition of soil organic matter (SOM) promotes R_min_ [19,20,21]. On the other hand, environmental factors can indirectly influence R_min_ by shaping microbial communities. For instance, soil moisture, soil temperature and pH may significantly affect soil bacterial abundances [22,23]. By influencing the activities of the soil microbiota, particularly their enzymatic activities, edaphic environmental factors play important roles in reshaping the microbial community [24,25]. The change of soil microbial biomass or microbial community is able to regulate soil N mineralization [26,27]. Variations in soil microbial biomass were reported to have a profound impact on N mineralization [28,29]. Besides, previous studies have demonstrated that the abundance of some key species in the soil ecosystem determined the cycling of N [30,31,32].

It is well established that soil microbial assemblages play crucial roles in regulating N cycling [33,34,35,36]. Given that functional genes can indicate microbial metabolic potential, it is important to understand the role of functional genes in regulating soil N cycling [37]. Previous studies have identified the key genes of the soil N mineralization pathway. For example, functional genes like *gdh* and *ureC* were verified as crucial genes in the soil N mineralization pathway [14,38,39,40]. Furthermore, Dai et al. (2020) reported a positive impact of *amoA* on nitrification processes [35]. Moreover, the role of functional genes as an assembly in determining N cycling has also been tested [41]. Compelling evidence regarding the crucial role played by soil properties and microbial assemblages in determining N cycling exists; however, the understanding of the means and pathways by which these attributes contribute to promoting soil N mineralization is still lacking [35]. Specifically, from the perspective of determining the N mineralization in forest soils, the relative importance of microbial community assemblages and functional genes assemblages on soil N mineralization has not yet been sufficiently established.

The boreal forest ecosystem is acknowledged as N-limited, in which accessible N serves multiple functions [42,43]. It is therefore evident that an understanding of the relationship between the soil N cycling, the soil microbial community and soil properties is essential for the effective regulation of forest growth, biodiversity and ecological function in this region [44]. However, the research on N mineralization in boreal forests is not yet comprehensive. Hence, investigating the interactive effects and relationships between soil N mineralization and its influencing factors in boreal forests remains important [44]. Based on the previous literature on the influencing factors of N mineralization, we found that functional genes represent a more accurate indicator for elucidating the processes of soil nitrogen mineralization, even when compared to the role of microbial community diversity [26,28,45]. Consequently, we hypothesized that functional genes not only offer superior predictive power but also provide a more comprehensive understanding of the pathways and underlying drivers that influence N mineralization. In the current study, the soil properties, net R_min_, and microbial assemblages (microbial community assemblages and functional genes assemblages) related to soil N mineralization in five typical forests were investigated to better understand the variation of soil N mineralization and its controlling factors in boreal forests. Partial least squares path modeling (PLS-PM) was conducted to evaluate the complex relationships between the variables of soil biotic and abiotic parameters involved in soil N mineralization, so as to further explore how influencing factors affect N transformations. Specific objectives of this study were threefold: (1) to elucidate the direct and indirect effects and contributions of soil properties and microbial assemblages on soil N mineralization; (2) to identify the factors driving soil N mineralization in boreal forests, and (3) to demonstrate that, compared to microbial community assemblages, functional genes assemblages serve as a more precise indicator for regulating soil N mineralization.

## 2. Materials and Methods

### 2.1. Study Site

**Figure 1:**
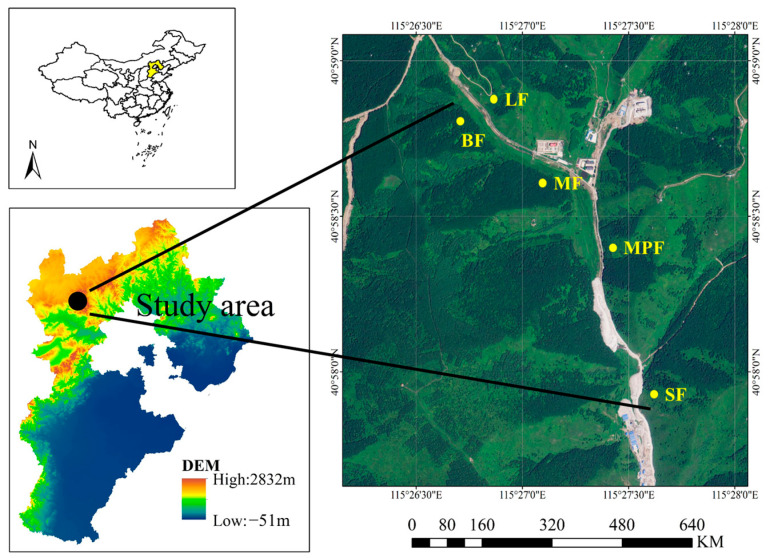
Location of the study site and sampling scheme.

The study was conducted in a mountainous area located in the Chongli District of northwestern Hebei Province, China (115.4604344 N, 41.0265855 E) (Figure 1). The elevation of the study area ranges from 1797 m to 2003 m, and the climate is typical of East Asia, with a continental monsoon pattern. Summers are cool and humid, while winters are cold [46]. The mean annual temperature for the region is 3.7 °C, and the average annual rainfall is approximately 490 mm. Most forests here were dominated by *Betula platyphylla* (BF), *Larix principis-rupprechtii* (LF), *Picea asperata* (SF) and *Pinus sylvestris* var. *mongolica* (MPF) [46]. The other forests were a mix of *Betula platyphylla* and *Larix gmelinii* (MF), with *Betula platyphylla* accounting for approximately 89.5% and *Larix gmelinii* accounting for approximately 10%. BF was classified as secondary forest, while LF, SF and MPF were artificially planted species. In total, there were 553 species of terrestrial plants in 301 genera of 80 families in the forests of this core area (Chongli District of Zhangjiakou Municipal People’s Government; www.zjkcl.gov.cn, accessed on 1 July 2024). The forest soil is comprised predominantly of chestnut soil with a minor amount of black soil. Table S1 tabulates a summary of the basic characteristics of these forest ecosystems, including the latitudes and longitudes of the experimental sites.

### 2.2. Experimental Design and Field Sampling

**Figure 2:**
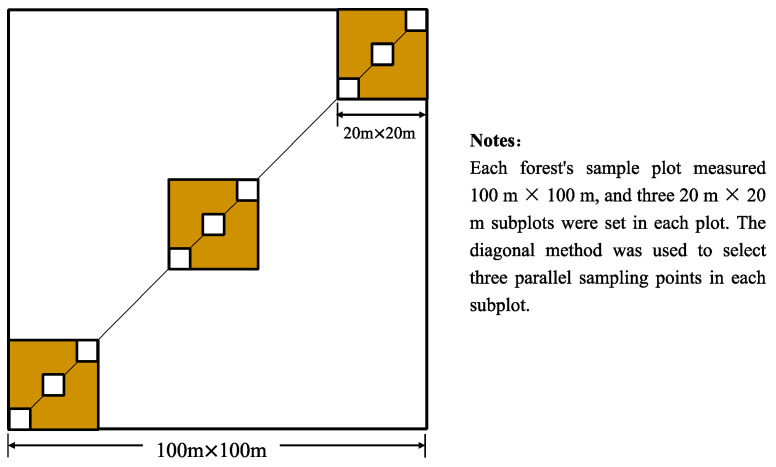
Field sampling scheme.

Five typical forest ecosystems (LF, BF, MF, SF and MPF) were studied. In each of them, an area of 100 m (length) × 100 m (width) was selected, and three 20 m × 20 m plots were established within each area. Further, three sampling points were selected in each plot using the diagonal method (Figure 2). Between the end of July 2019 and the end of April 2020, measurements of net soil N mineralization were conducted at each sampling point (sampling once every three months) using the in situ incubation method with PVC cores, in accordance with the methodology outlined by Raison et al. [47]. At each sampling point, two PVC cores (6 cm in diameter and 20 cm in height) were inserted to a depth of 10 cm, which was identified as the most active soil layer (0–10 cm) [48]. Soil temperature was measured in situ at a depth of 5 cm using a temperature probe. At each sampling point, one of the PVC cores (the initial sample) was immediately removed from the soil for initial inorganic N analyses. The other PVC core was left in the field for approximately three months, covered with a permeable plastic film on top and gauze at the bottom to manage water segregation and allow gas exchange [49]. Upon completion of the incubation period, the samples were retrieved and transported to the laboratory for analysis. The samples from the three parallel sampling points were thoroughly mixed, sieved through a 2 mm mesh, and processed as final samples. These mixed samples were used to measure soil physicochemical properties (see next section for details). In addition to the PVC core samples, an additional soil sample was collected near each sampling point after removing the surface litter for the measurement of microbial functional genes [18]. There were in total 180 samples collected to compare net N mineralization among forest types.

### 2.3. Soil Physicochemical Properties

For determinations of soil NH_4_^+^ and NO_3_^−^ concentrations, samples were extracted with a 2 mol L^−1^ KCl solution and measured using a continuous flow analyzer (AutoAnalyzer3, Bran+Luebbe, Norderstedt, Germany) [50]. The soil net ammonification rate (R_amm_), nitrification rate (R_nit_), and net R_min_ were determined by calculating the difference in the concentrations of soil NH_4_^+^-N, NO_3_^−^-N, and mineral N (NH_4_^+^-N + NO_3_^−^-N) between initial and post-incubation sample time points [49]. Soil moisture was measured after drying the subsamples at 105 °C for 24 h. Soil pH was determined using a glass electrode meter in a 1:2.5 soil/water solution (H_2_O). SOM content was measured by the wet oxidation method with K_2_Cr_2_O_7_ and H_2_SO_4_ [51]. Total N (TN) content was determined using the Kjeldahl method [52], and total phosphorus (TP) was measured by wet digestion with HClO_4_–H_2_SO_4_ and determination by UV spectrophotometer (UNICO, Shanghai, China) [53]. The cation exchange capacity (CEC) was determined by the ammonium saturation method [54]. The soil physicochemical properties are given in Table S2.

### 2.4. Measurement of Soil Microbial Functional Genes Involved in N Mineralization

The microbial functional genes were characterized using GeoChip 5.0 and in accordance with standard protocols [55]. The analysis was carried out by Guangdong Magigene Biotechnology Co., Ltd. (Guangzhou, China). Briefly, genomic DNA was extracted using a PowerSoil^®^ DNA Isolation Kit (MoBio Inc., Carlsbad, CA, USA). Prior to hybridization, DNA was labelled and suspended in a hybridization solution for subsequent analysis on a MAUI station (MAUI, BioMicro Systems, Salt Lake City, UT, USA). GeoChip microarrays were then scanned by a NimbleGen MS200 scanner (Roche, Madison, WI, USA). Next, the signal intensities were quantified and processed using a previously described data analysis procedure [41]. Lastly, the extracted data were subjected to analysis using the GeoChip data analysis pipeline (http://ieg.ou.edu/microarray/, accessed on 1 July 2024) [55,56]. As the objective of the present research was to investigate soil N mineralization, the gene families primarily involved in ammonification and nitrification (namely, *gdh*, *glnA_fungi*, *ureC*, *amoA* and *hao*) were the focus of our analysis.

### 2.5. Statistical Analysis

Before the analysis of variance, the normality and homogeneity was tested. One-way analysis of variance (ANOVA) followed by LSD (with three replicates) was performed to evaluate the effects of forest types on soil R_min_. Redundancy analysis (RDA) was performed to evaluate the influence of soil properties and microbial assemblages on R_min_ (Canoco 5.0). To test the importance of microbial community assemblages (α-diversity, Shannon and Richness) and functional genes assemblages on soil N mineralization, a linear mixed model was constructed using R (version 4.1.3) package lme4. Therefore, Akaike Information Criterion (AIC) value was used to determine the best model between microbial functional genes assemblages and microbial community assemblages (α-diversity). The above analyses were performed in SPSS 26.0 (SPSS Inc., Chicago, IL, USA) at the significance level of *p* < 0.05.

Partial least squares path modeling (PLS-PM) was used to explore the standardized direct and indirect effects of soil properties, microbial functional genes assemblages and microbial community assemblages on soil N mineralization. PLS-PM is a powerful statistical method for studying interactive relationships between observed and latent variables [57], and it is widely used to explain and predict relationships in multivariate data sets. In this study, the soil properties were further classified into edaphic environments (i.e., soil temperature, soil moisture and pH) and vegetation-derived substrates (i.e., SOM, TN, TP, CEC, NH_4_^+^ and NO_3_^−^). In the model, the indexes whose absolute standard loadings exceed 0.7 were selected. The goodness of fit (GoF) statistics were used to evaluate the models of different structures. The R package ‘plspm’ was used to build the final PLS-PM model.

## 3. Results

### 3.1. Soil Net N Mineralization

**Figure 3:**
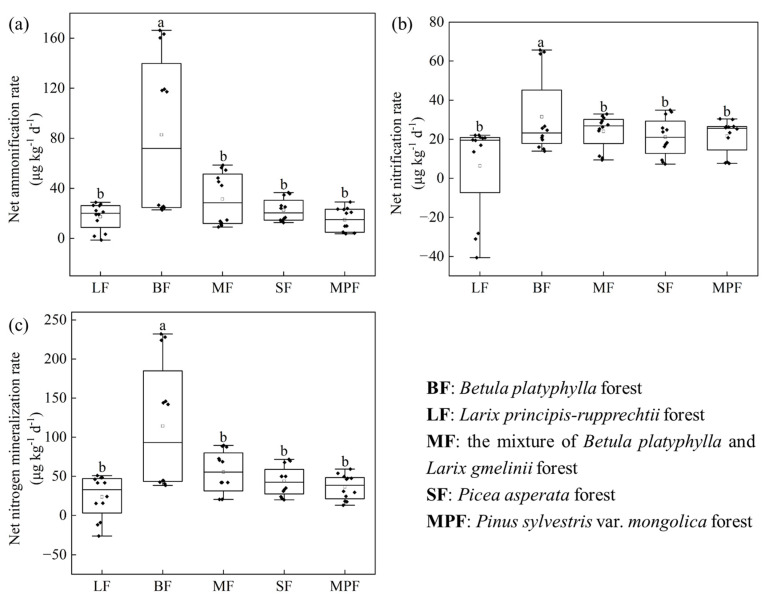
The soil net R_amm_ (**a**), net R_nit_ (**b**) and net R_min_ (**c**) in the five forests. The square in the box indicates the mean value. Lowercase letters indicate significant differences among the five forests (LSD, *p* < 0.05).

Soil net R_amm_, R_nit_ and R_min_ differed significantly among different forest types (*p* < 0.05, Figure 3). The average soil R_amm_ (82.67 µg kg^−1^ d^−1^), R_nit_ (31.48 µg kg^−1^ d^−1^) and R_min_ (114.15 µg kg^−1^ d^−1^) in BF were significantly higher than that in other forests (*p* < 0.05). During the entire study period, the range of soil net R_amm_, R_nit_ and R_min_ in BF were also largest (22.74–166.25 µg kg^−1^ d^−1^, 13.87–65.66 µg kg^−1^ d^−1^, 38.38–227.92 µg kg^−1^ d^−1^). In other forests, the soil net R_amm_, R_nit_ and R_min_ were relatively concentrated.

### 3.2. Relationships among Soil Factors, Microbial Community Diversity and Soil N Mineralization

**Figure 4:**
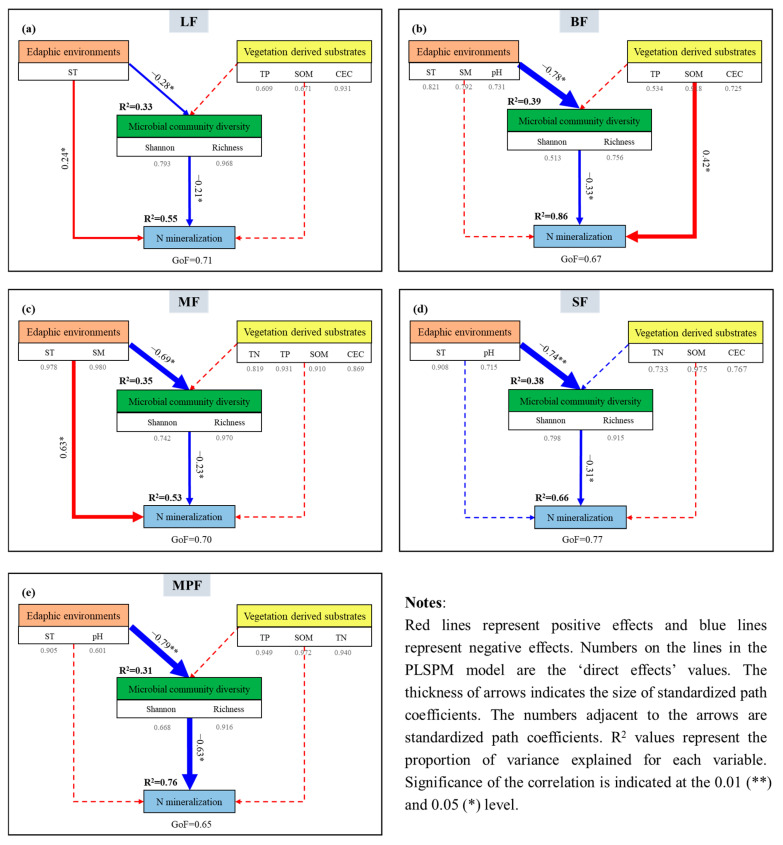
PLS-PM depicting the influence of edaphic environments and substrates on soil microbial community and N mineralization in the five forests. Notes: Results of model fitting: (**a**) LF: GoF = 0.71; (**b**) BF: GoF = 0.67; (**c**) MF: GoF = 0.70; (**d**) SF: GoF = 0.77; (**e**) MPF: GoF =0.65. Edaphic environments: soil temperature (ST), soil moisture (SM), pH; vegetation-derived substrates: TN (total nitrogen), TP (total phosphorus), SOM (soil organic matter), CEC (cation exchange capacity), NH_4_^+^, NO_3_^−^; microbial community diversity: Shannon, Richness.

The results of RDA indicated that soil net R_amm_ and R_nit_ were closely correlated to soil factors and microbial assemblages (including microbial community diversity) (Figure S3). The PLS-PM analyses were performed to characterize the intricate association among edaphic environments, vegetation-derived substrates, soil microbial community assemblages and N mineralization (Figure 4). Similar to the results of the RDA, the PLS-PM also showed that soil microbial assemblages strongly affected soil N mineralization in different forests (Figure 4 and Figure S3). As shown in Figure 4a–e, the PLS-PM accounted for 53–86% of the variations of soil N mineralization in different forests. Among soil microbial assemblages, edaphic environments significantly impacted the microbial assemblages and accounted for more than 30% of the variation in microbial community diversity. However, the direct effect of substrates derived from vegetation on microbial community diversity was weak in all five forests. Furthermore, in LF and MF, the soil environment not only indirectly increased soil N mineralization via microbial factors, but also directly and positively influenced soil N mineralization (Figure 4a,c). In general, different forests showed distinct paths (Figure 4), but all of them highlight the potential of soil microbes in explaining the variations of soil N among different forests.

### 3.3. Factors Driving Soil N Mineralization

**Figure 5:**
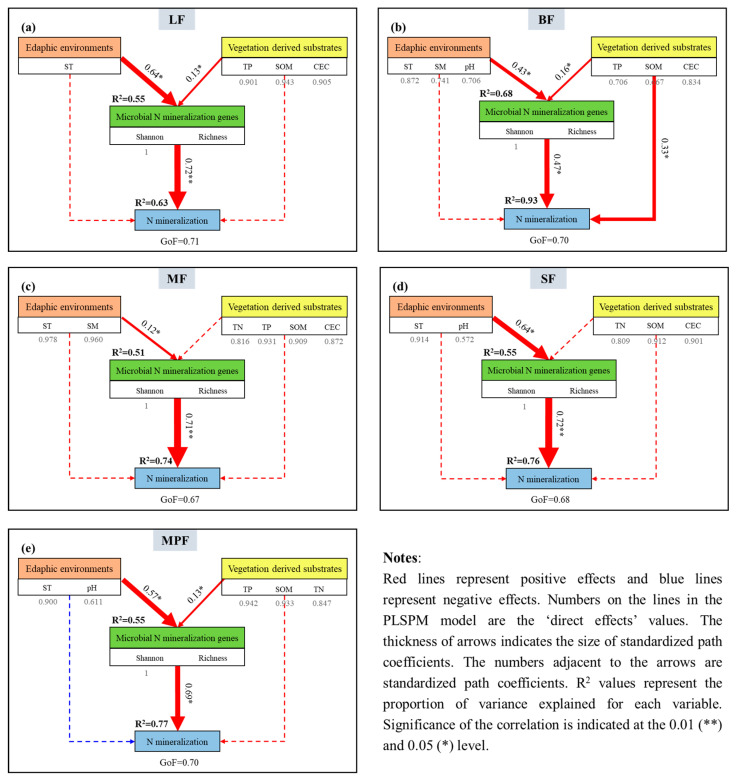
PLS-PM depicting the influence of edaphic environments and substrates on soil microbial N mineralization genes and N mineralization in the five forests. Notes: Results of model fitting: (**a**) LF: GoF = 0.71; (**b**) BF: GoF = 0.70; (**c**) MF: GoF = 0.67; (**d**) SF: GoF = 0.68; (**e**) MPF: GoF = 0.70. Edaphic environments: soil temperature (ST), soil moisture (SM), pH; vegetation-derived substrates: TN (total nitrogen), TP (total phosphorus), SOM (soil organic matter), CEC (cation exchange capacity), NH_4_^+^, NO_3_^−^; microbial community diversity: Shannon, Richness.

RDA results revealed the significant relationships among soil net R_amm_ and R_nit_ and influencing factors (including microbial functional genes assemblages) (Figure S3). Soil microbial functional genes assemblages had a comparatively stronger impact than community on soil N mineralization in all five forests (Figure 5 and Figure S3). In Figure 5 a–e, it was found that the results of PLS-PM accounted for 63–98% of the variations in soil N mineralization across various forests, indicating that the interpretation approaches of soil N mineralization in different forests may not be entirely consistent. Specifically, soil microbial functional genes frequently had a positive effect on soil N mineralization (Figure 5 and Table S4). Among soil properties, edaphic environments demonstrated strong influence on microbial genes assemblages; however, vegetation-derived substrates only impacted the microbial genes assemblages directly in LF, BF and MPF (Figure 5a,b,e). In addition, edaphic factors directly enhanced soil microbial genes assemblages across various forests and indirectly influenced soil N mineralization by impacting soil microbial properties (Figure 5). In general, soil temperature was the predominant soil environmental factor that indirectly influenced the level of soil N mineralization through its effect on microbial genes assemblages. Moreover, in BF, the substrates directly led to an increase in soil N mineralization, while this process was not observed in other types of forests (Figure 5b).

**Figure 6:**
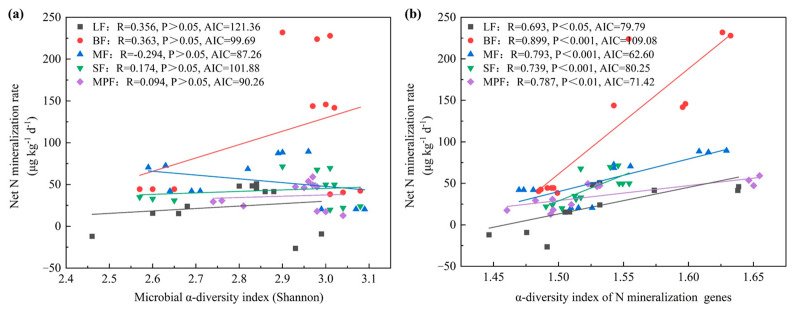
The results of the linear mixed model for soil N mineralization in the five forests. (**a**) Correlations for soil net R_min_ to microbial community diversity: Shannon, Richness; (**b**) correlations for net R_min_ to diversity index of microbial N mineralization genes.

In general, the microbial assemblages directly impacted soil N mineralization, and compared to microbial community assemblages, functional genes assemblages showed stronger effect with the “direct effects” values of 0.72, 0.47, 0.71, 0.72 and 0.69 for LF, BF, MF, SF and MPF, respectively. It was demonstrated that functional genes assemblages’ index outperformed the community assemblages’ index in the mixed effect models with a lower AIC value between the two microbial assemblages’ indexes (Table S3 and Figure 6). Additionally, functional genes assemblages accounted for a greater variation in soil N mineralization and the interpretation rates increased by 8%, 7%, 21%, 10% and 1% for LF, BF, MF, SF and MPF, respectively. Overall, N mineralization genes were the main factors influencing the variation of soil N mineralization (Figure 5), whereas in comparison with substrates, soil environmental factors played a greater role in driving microbial assemblages and thus indirectly controlling the net R_min_.

## 4. Discussion

### 4.1. Influencing Factors of Soil N Mineralization

Soil R_min_ among five forests during the whole study period showed significant differences (Figure 3), and the order was BF > MF > SF > MPF > LF. The results supported the previous findings that forest types altered soil N mineralization [27,41] which was largely due to the following mechanisms. Firstly, it was due to alterations in the composition of tree species and the quality of litter. Previous research has shown that conifer species typically produce low-quality litter material, characterized by lower nitrogen content and higher C:N ratios, resulting in slow litter decomposition. This, in turn, contributes to poor soil nutrient levels, thereby reducing the rate of N mineralization and nitrification [58,59]. Furthermore, broadleaf species may enhance N transformation due to lower lignin:N and C:N ratios in the litter [29]. Secondly, based on the results of RDA, soil R_min_ can be attributed to the changes of soil properties and microbial factors (Figure S3) in this study. There was a positive correlation between soil temperature/moisture and R_min_ (Table S5), which was consistent with earlier observations [60,61,62].

Moreover, soil microorganisms played a critical role in N transformations, which means that their abundance or biomass might be proportional to soil N mineralization [63,64,65,66]. Soil microbial assemblages directly regulated soil N mineralization, which was studied in terms of both structure and function. Soil microbial communities in this region were mainly bacteria (Figure S2), and soil microbial assemblages differed significantly among the forest types (Figure S3 and Table S6). In particular, the microbial assemblages’ indexes of LF were significantly lower than those of BF and MF (Figure S1 and Table S6). At the same time, it was found that the abundance of *Proteobacteria* and *Actinobacteria*, which dominated the soil bacterial community (Figure S2), was usually higher in BF and MF than in LF, SF and MPF. *Actinobacteria* were reported to play an essential role in breaking down refractory polymers [67], which helped to decompose low-quality organic matter and promoted N transformation in the forests. Therefore, the net R_min_ was higher in BF and MF that contained more *Actinobacteria*, which was consistent with previous studies. These results indicated that soil N mineralization and N cycling were generally associated with variations in soil microorganisms [26,28].

Soil microbial functional gene assemblages played an important role in directly regulating N mineralization, which can explain the greater variation in soil N mineralization than community diversity (Figure 5). It was found that the observed pattern of net N mineralization rates was correlated to N cycling genes (Figure S3 and Table S4), and further analysis showed that the contribution was greater than that of community diversity (Figure 4 and Figure 5). Compared with microbial community assemblages, functional genes assemblages had stronger influences on soil N mineralization (positive, 0.72, 0.47, 0.71, 0.72 and 0.69 for LF, BF, MF, SF and MPF, respectively), and with the explanation rates increased. These results showed that microbial functional traits can influence soil ecological functions more than structural traits [68]. In this research, microbial functional genes were important factors to regulate net soil N mineralization.

Nevertheless, Nakayama et al. (2021) suggested that micro-subsets sharing similar niches may be important factors affecting the net soil N mineralization potential, instead of functional genes [69]. The controversy may be linked to the intricate interplay between ecosystem processes and microbial traits. This study focused on individual functions rather than the adaptability of the ecosystem. Therefore, versatility of functional genes was more important to individual function than microbial diversity and can be used as an indicator to measure the impact of community on soil N mineralization.

### 4.2. Determinants of Soil N Mineralization

According to the metabolic theory of ecology, Liang et al. (2020) proposed that substrate and temperature play important roles in the formation of functional microbial communities [70]. Environmental factors such as soil moisture, soil temperature, and pH may significantly impact soil bacterial abundance [22,23]. In this regard, the reports of Ding (2015) and Scarlett (2021) also identified the critical role of soil environmental factors like pH and temperature in shaping the distribution of functional genes in oak forests and forest ecosystems [36,38], which was similar to this research. The results of this study indicated a significant correlation between soil properties and microbial genes in soil N mineralization, especially soil moisture and temperature (Table S7). Consequently, it can be concluded that soil environmental factors directly affected microorganisms. Soil environmental factors had an indirect impact on soil N mineralization by affecting functional N cycling genes (Figure 5). Moreover, we have identified five typical soil N mineralization pathways in boreal forests through comprehensive analysis.

In summary, through analyzing the soil N mineralization processes in five boreal forests, it was determined that microbial genes were the dominant factor in net soil N mineralization. The findings verified that soil microbial assemblages directly impact N mineralization, whilst soil properties control soil N mineralization indirectly by affecting microbial assemblages. The change in soil microorganisms was the key link between soil properties and soil inorganic N. Overall, this study, in conjunction with previous observations, provided evidence for a strong correlation between microbial functional attributes and ecosystem functions, indicating that functional genes were more sensitive than microbial structural attributes in predicting changes in soil functions such as soil N mineralization [71]. The highly significant correlation between soil functions and functional genes highlighted the crucial role played by soil microorganisms in maintaining multiple soil functionalities [72]. This research suggested that the assembly of functional genes was a more appropriate parameter for indicating changes in soil N mineralization compared to the assembly of microbial communities. To expand its application, the utilization of the abundance and quantity of functional genes, which can provide a more precise measurement of soil N mineralization in boreal forests, was proposed (Figure S4). This study highlighted a novel instance, where the functional traits of microorganisms showed greater impacts on soil N mineralization than community assemblages in boreal forests. In other words, microbial functional assemblages provided more accurate information that can be used for ecosystem and biogeochemical modelling, as well as protection and management policies [73]. In addition, introducing a gene-centered view into the relationship between soil factors and soil N mineralization will facilitate a deeper comprehension of the effects of influential factors on ecosystem functions. This will contribute effectively to the protection of species diversity and restoration of the fragile ecosystems of boreal forests.

## 5. Conclusions

In this study, the pattern of net soil N mineralization among five boreal forests was characterized, and the contributions of microbial and edaphic factors to processes contributing to soil N mineralization as well as the direct and indirect effects between edaphic environments, vegetation-derived substrates, soil microbial assemblages and soil N mineralization were ascertained. Differences in the influencing factors between the five forests and variations in the interpretation of the soil N mineralization pathways across these forests were discovered. The PLS-PM results indicated that variations in soil N mineralization were mainly explained by soil factors and functional genes, accounting for 63–93% of the variability. In general, it was revealed that soil environmental factors were identified as playing regulatory roles in determining the soil functional N mineralization genes assemblages. Compared with microbial community assemblages (Shannon and Richness), functional genes assemblages played a crucial role in regulating N mineralization and are the primary drivers of soil N mineralization. In conclusion, the findings of the present study offered valuable insights into predicting the impact of global changes on N cycling genes and their ecological functions which could additionally support the conservation and restoration of forest ecosystems.

## Data Availability

The original contributions presented in the study are included in the article/supplementary material, further inquiries can be directed to the corresponding author.

## Supplementary Materials

The following supporting information can be downloaded at: https://www.mdpi.com/article/10.3390/microorganisms12081577/s1, Figure S1: Box plots of microbial properties in five forests; Figure S2: Abundances of soil microbial groups at the phylum level in each plot of the five forests; Figure S3: RDA analysis showing the association of net R_amm_ and net R_nit_ to soil properties and soil N mineralization microbial properties for the samples from different forests; Figure S4: Partial Least Squares Path Modeling; Table S1: The basic characteristics of the five typical forest ecosystems; Table S2: Soil properties in the five forests; Table S3: The results of linear mixed model for soil N mineralization; Table S4: Correlation between soil net R_amm_, R_nit_, R_min_ and microbial properties in the five forests; Table S5: Correlation between soil net R_amm_, R_nit_, R_min_ and soil properties in the five forests; Table S6: Summary of one-way ANOVA results for the effects of forest type on the abundance of *Proteobacteria* and *Actinobacteria* among the five different forests (Turkey’s test); Table S7: Correlation between microbial properties and soil properties in the five forests.

## Abbreviations

R_min_, rates of soil nitrogen mineralization; R_amm_, rates of soil ammonification; R_nit_, rates of soil nitrification; SOM, soil organic matter; TN, total nitrogen; TP, total phosphorus; CEC, cation exchange capacity; PCA, principal component analysis; RDA, redundancy analysis; PLS-PM, partial least squares path modeling; LF, *Larix principis-rupprechtii* forest; BF, *Betula platyphylla* forest; MF, mixed forest of *Larix principis-rupprechtii* and *Betula platyphylla*; SF, *Picea asperata* forest; MPF, *Pinus sylvestris* var. *mongolica* forest.
