# Supplementary material for: Diversity of Microbial Functional Genes Promotes Soil Nitrogen Mineralization in Boreal Forests
Source: Microorganisms. 2024 Aug 2;12(8):1577. doi: 10.3390/microorganisms12081577 (PMC11355967; doi:10.3390/microorganisms12081577)
Supplement: Supplementary file 1 [file microorganisms-12-01577-s001.zip › microorganisms-3133675-supplementary.pdf]

Supplementary materials

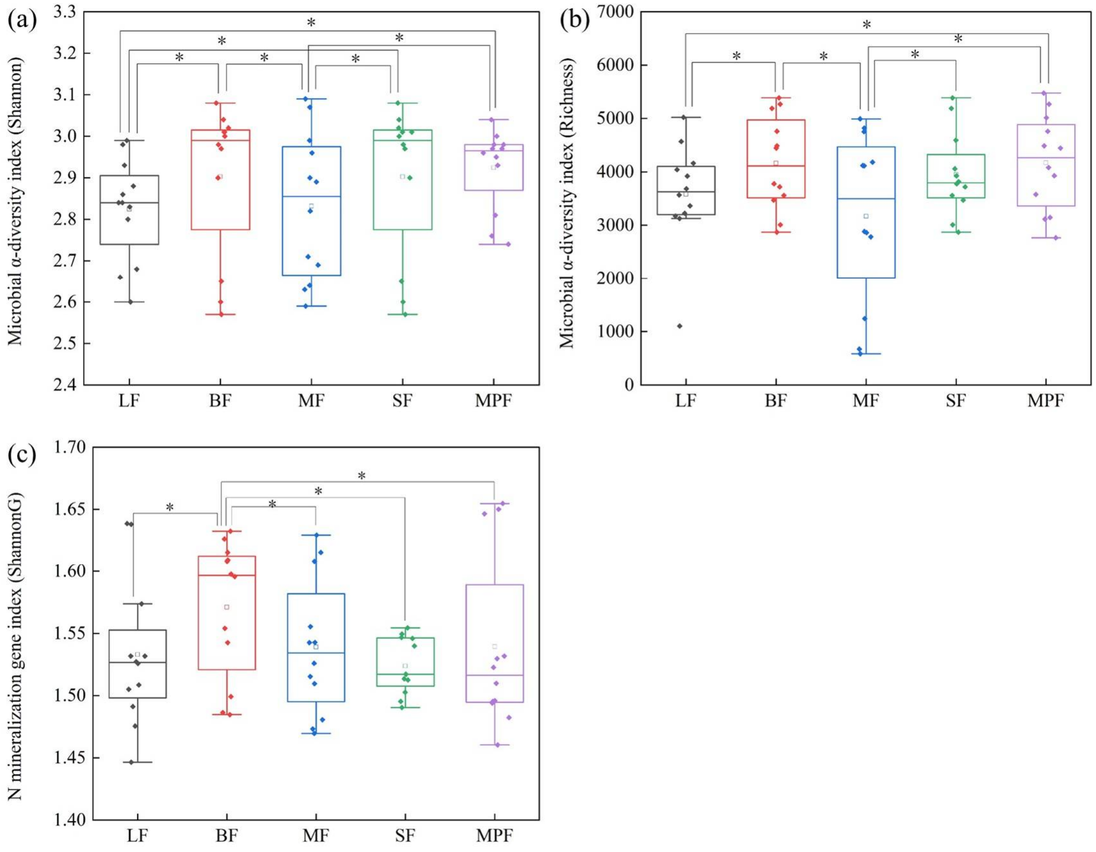

Figure S1. Box plots of microbial properties in five forests, (a) microbial diversity of Shannon index, (b) microbial diversity of Richness, and (c) N mineralization gene Shannon index. The bar is the interquartile range, the upper whisker line is the maximum value, and the lower is the minimum value. The horizontal line in the bars is the median value, the square in the bars is the mean value. The * over the bars indicate significant difference between forests (HSD, $P < 0.05$). Soil N mineralization microbial α-diversity in the five forests were determined for each sample using the Richness and Shannon index. One-way ANOVA with a post hoc HSD test was used to compare effects of forest types on microbial properties over the study period.

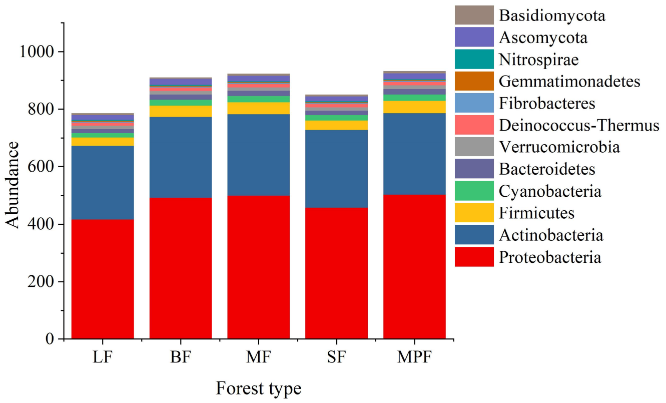

Figure S2. Abundances of soil microbial groups at the phylum level in each plot of the five forests.

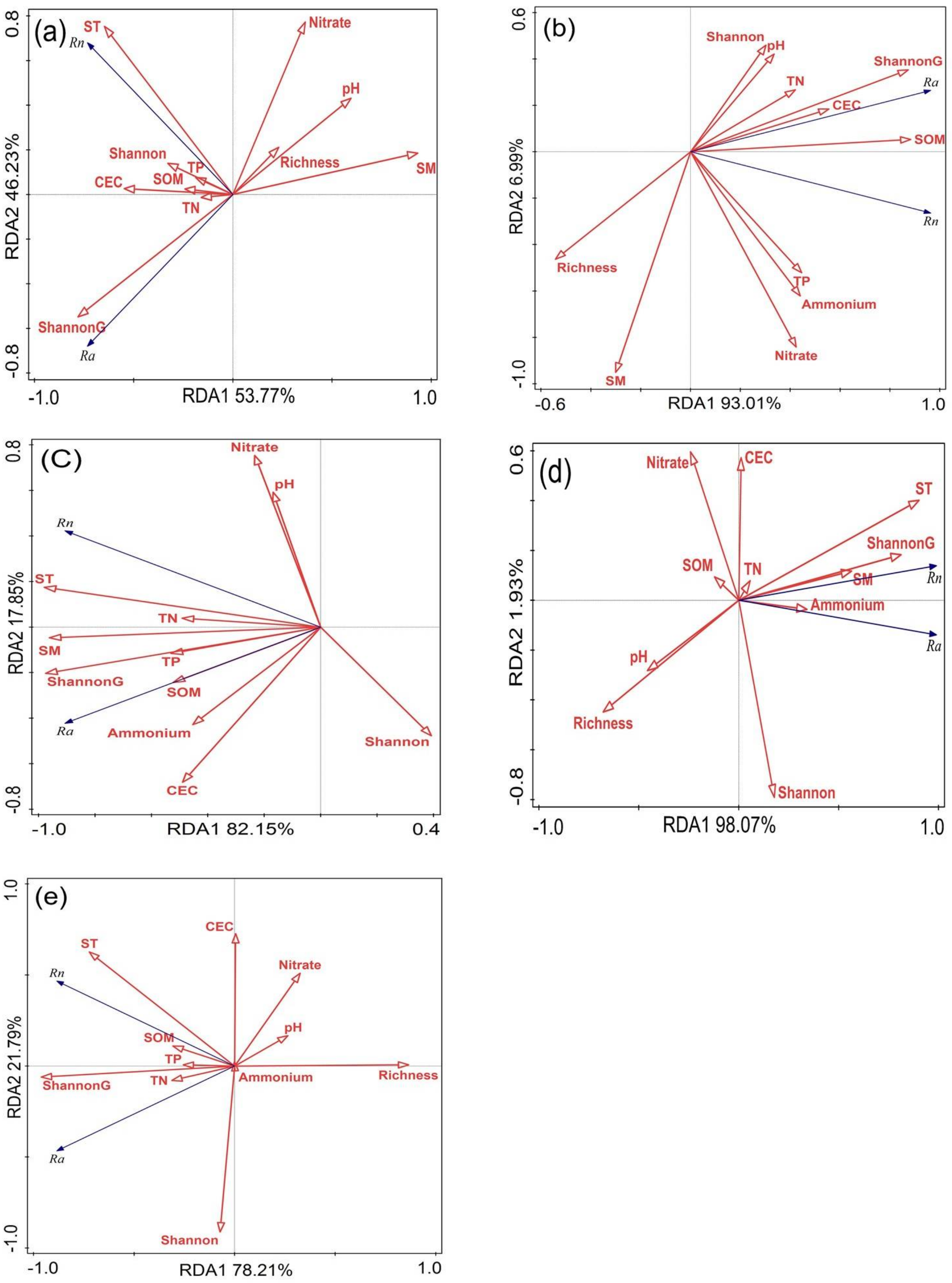

Figure S3. RDA analysis showing the association of net R_{amm} and net R_{nit} to soil properties and soil N mineralization microbial properties for the samples from different forests. (a) LF; (b) BF; (c) MF; (d) SF; (e) MPF. Notes: Richness and Shannon, diversity index of microbial community; ShannonG, diversity index of N mineralization genes.

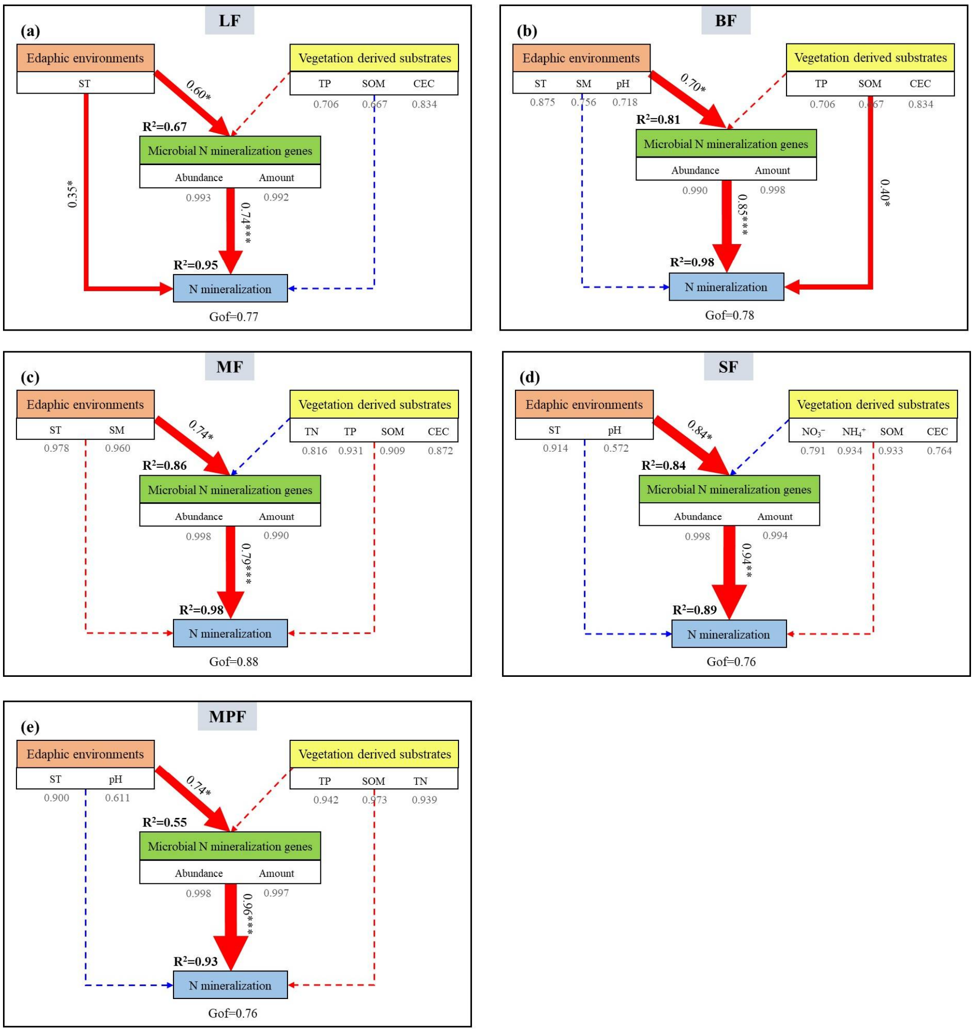

Figure S4. Partial Least Squares Path Modeling. Notes: Results of model fitting: (a) LF: Gof=0.77; (b) BF: Gof=0.78; (c) MF: Gof=0.88; (d) SF: Gof=0.76; (e) MPF: Gof=0.76. Edaphic environments: ST, SM, pH; Vegetation derived substrates: TN, TP, SOM, CEC, NH_4^+, NO_3^-; Microbial N mineralization genes: Abundance and amount. Red lines represent positive effects and blue lines represent negative effect. Numbers on the lines in the PLS-PM model are the 'direct effects' values. The thickness of arrows indicates the size of standardized path coefficients. The numbers adjacent to the arrows are standardized path coefficients. R^2 values represent the proportion of variance explained for each variable. Significance of the correlation is indicated at the 0.001 (***), 0.01 (**), and 0.05 (*) level.

Table S1. The basic characteristics of the five typical forest ecosystems.

Stands	Slop aspect	Longitude and latitude	Altitude m·a·s·l[a]	Stand density (trees/ha)	Average height of trees (m)	Diameter at breast height (cm)
LF	Southwest	115°26'58"E 40°58'53"N	1985	1138	7.6	10.29
BF	Northeast	115°26'37"E 40°58'48"N	1962	693	9.8	17.2
MF	Northeast	115°27'51"E 40°58'37"N	1904	2201	9.9	13.4
SF	Southwest	115°27'41"E 40°57'43"N	1823	650	3.2	5.6
MPF	Southwest	115°27'55"E 40°57'26"N	1860	630	1.8	5.3

[a]: Meters above sea level.

Notes: *Larix principis-rupprechtii* forest (LF), *Betula platyphylla* forest (BF), mixed forest of *Larix principis-rupprechtii* and *Betula platyphylla* (MF), *Picea asperata* forest (SF) and *Pinus sylvestris var. mongolica* forest (MPF).

Table S2. Soil properties in the five forests.

Forest	LF	BF	MF	SF	MPF
**ST °C**	9.76±0.18ab	6.5±0.18b	4.14±0.08b	9.42±0.16ab	11.42±0.25a
**SM**	37.61±0.55ab	42.22±0.43a	36.21±0.34ab	33.83±0.36b	35.92±0.45ab
**pH**	6.92±0.11a	6.85±0.10a	6.61±0.26b	6.92±0.11a	6.91±0.08a
**TN g/kg**	2.79±0.56b	3.8±0.23ab	3.97±0.45a	2.78±0.38b	3.18±0.51ab
**TP g/kg**	0.78±0.07ab	0.69±0.06ab	0.87±0.08a	0.65±0.06b	0.67±0.078b
**SOM g/kg**	58.59±11.84b	86.42±10.55a	87.22±10.46a	57.92±15.38b	64.72±17.77b
**CEC cmol (+)/kg**	30.51±3.34ab	34.69±4.74a	34.41±3.37a	26.21±4.76b	30.77±5.77a
**NH_4^+ mg kg^{-1}**	3.59±0.30b	7.75±0.88a	5.78±0.51ab	4.66±0.40b	3.70±0.48b
**NO_3^- mg kg^{-1}**	10.91±0.52b	10.48±1.45b	13.30±1.52a	12.45±1.20ab	10.77±0.96b

Notes: Values are presented as means ±SE (n=9). Means with no letter in common are significantly different (HSD, α=5%). Abbreviations: ST, soil temperature; SM, soil moisture; CEC, cation exchange capacity; TN, total nitrogen; TP, total phosphorus; SOM, soil organic matter. One-way ANOVA with a post hoc HSD test was used to compare effects of forest types on soil properties over the study period.

Table S3. The results of linear mixed model for soil N mineralization.

Forest type	Factor	AIC	P
**LF**	N mineralization genes diversity	**79.793**	**<0.05**
	Microbial community diversity	99.691	>0.05
**BF**	N mineralization genes diversity	**109.084**	**<0.001**
	Microbial community diversity	121.364	<0.001
**MF**	N mineralization genes diversity	**62.601**	**<0.001**
	Microbial community diversity	87.259	<0.05
**SF**	N mineralization genes diversity	**80.251**	**<0.001**
	Microbial community diversity	101.879	<0.001
**MPF**	N mineralization genes diversity	**71.416**	**<0.01**
	Microbial community diversity	90.258	<0.05

Table S4. Correlation between soil net R_{amm}, R_{nit}, R_{min} and microbial properties in the five forests.

**Forests**		**Richness**	**Shannon**	**ShannonG**	**Amount**	**Abundance**
	R_{amm}	-0.313	0.146	0.332	0.565	.604*
**LF**	R_{nit}	-0.026	0.335	.631*	.790**	.794**
	R_{min}	-.586*	0.556	.693*	.925**	.944**
	R_{amm}	-.645*	0.413	.916**	.928**	.951**
**BF**	R_{nit}	-0.401	0.17	.753**	.770**	.767**
	R_{min}	-.601*	0.363	.900**	.913**	.930**
	R_{amm}	-.766**	0.154	.882**	.965**	.984**
**MF**	R_{nit}	-0.475	0.557	0.367	.746**	.736**
	R_{min}	-.738**	.594*	.793**	.976**	.987**
	R_{amm}	-.610*	0.286	.693*	.716**	.761**
**SF**	R_{nit}	-.735**	0.066	.768**	.770**	.800**
	R_{min}	-.681*	0.174	.739**	.751**	.789**
	R_{amm}	-.767**	0.489	.797**	.875**	.907**
**MPF**	R_{nit}	-.761**	0.361	.587*	.792**	.759**
	R_{min}	-.864**	0.594*	.787**	.944**	.946**

Notes: Pearson correlation was used to examine the correlations between soil net R_{amm}, R_{nit}, R_{min} and microbial properties. *, $P < 0.05$; **, $P < 0.01$. R, Pearson's correlation coefficient. Abbreviations: ST, soil temperature; SM, soil moisture; CEC, cation exchange capacity; TN, total nitrogen; TP, total phosphorus; SOM, soil organic matter.

Table S5. Correlation between soil net R_{amm}, R_{nit}, R_{min} and soil properties in the five forests.

Forests		ST	SM	pH	TN	TP	SOM	CEC	NH_4^+	NO_3^-
**LF**	R_{amm}	-0.037	0.121	-.810**	0.126	0.086	0.161	0.384	0.575	-.791**
	R_{nit}	.986**	-0.292	-0.558	0.107	0.189	0.193	0.419	0.041	0.25
	R_{min}	.865**	-0.213	-.523	0.145	0.202	0.235	0.523	0.261	-0.085
**BF**	R_{amm}	.843**	0.435	-0.341	0.476	0.292	.866**	.584*	0.26	0.186
	R_{nit}	.750**	0.212	-0.138	0.334	0.567	.838**	0.486	.589*	.631*
	R_{min}	.842**	.639*	-.647*	0.453	.589*	.882**	0.575	0.35	0.303
**MF**	R_{amm}	.814**	0.105	0.475	0.429	0.53	.576*	.732**	.592*	0.257
	R_{nit}	.962**	0.471	0.141	0.462	0.432	0.371	0.156	0.23	0.474
	R_{min}	.929**	.668*	0.261	0.475	0.543	0.559	.611*	0.527	0.347
**SF**	R_{amm}	.838**	-0.46	-0.541	0.046	0.266	-0.133	-0.068	0.343	-0.322
	R_{nit}	.949**	-0.409	-0.038	0.068	0.246	-0.107	0.091	0.333	-0.156
	R_{min}	.905**	-0.438	-.629*	0.058	0.258	-0.121	0.015	0.341	-0.638*
**MPF**	R_{amm}	0.347	-0.206	0.315	0.314	0.224	0.22	-0.343	-0.01	-0.46
	R_{nit}	.932**	-0.236	0.159	0.24	0.23	0.323	0.336	0.012	0.033
	R_{min}	.708**	-0.249	-.650*	0.315	0.256	0.305	-0.022	0.001	-0.254

Notes: Pearson correlation was used to examine the correlations between soil net R_{amm}, R_{nit}, R_{min} and soil properties. *, $P < 0.05$; **, $P < 0.01$; R, Pearson's correlation coefficient. Abbreviations: ST, soil temperature; SM, soil moisture; CEC, cation exchange capacity; TN, total nitrogen; TP, total phosphorus; SOM, soil organic matter.

Table S6. Summary of one-way ANOVA results for the effects of forest type on the abundance of Proteobacteria and Actinobacteria among the five different forests (Turkey's test).

Variables	Forest Type
**Proteobacteria**	<0.05
**Actinobacteria**	<0.05

Table S7. Correlation between microbial properties and soil properties in the five forests.

Forests		ST	SM	pH	TN	TP	SOM	CEC	NH_4^+	NO_3^-
	**Shannon**	-0.345	-0.359	0.058	-0.298	-0.145	-0.04	0.539	-0.047	-0.131
	**Richness**	-.622*	-0.262	0.194	-0.432	-0.223	-0.178	0.267	-0.351	0.262
	**ShannonG**	.607*	-.586**	-0.315	0.003	-0.568	.583*	0.517	0.226	-0.365
**LF**	**Prote**	-.812**	.807**	0.153	-0.194	-0.311	-0.322	-.641*	-0.272	0.093
	**Actin**	-.753**	.815**	0.108	-0.208	-0.318	-0.345	-.651*	-0.296	0.135
	**Amoun**	.745**	-.812**	-0.205	0.052	0.239	0.221	.626*	0.149	-0.175
	**Abund**	.743**	-.853**	-0.169	0.134	0.274	0.264	.658*	0.281	-0.205
	**Shannon**	-0.101	-.584*	0.222	0.556	-0.042	0.303	.855**	-.592*	-0.407
	**Richness**	-.863**	-0.561	-0.304	-0.128	0.111	-0.421	0.122	-0.229	0.057
	**ShannonG**	.793**	-0.589*	0.582*	0.383	0.133	.753**	0.469	0.215	0.112
**BF**	**Prote**	-.880**	0.56	-0.449	-0.508	-0.284	-.835**	-0.499	-0.313	-0.148
	**Actin**	-.862**	0.568	-0.435	-0.511	-0.283	-.845**	-0.549	-0.273	-0.152
	**Amoun**	.880**	-0.536	0.312	0.325	0.101	.741**	0.369	0.301	0.134
	**Abund**	.895**	-.588*	0.387	0.372	0.135	.777**	0.392	0.279	0.101
	**Shannon**	-0.425	-0.536	0.458	0.075	0.022	-0.007	0.331	-0.11	0.144
	**Richness**	-.761*	-.846**	-0.023	-0.133	-0.319	-0.41	-0.393	-.627*	0.187
	**ShannonG**	.596*	.593*	0.309	0.449	0.553	0.552	.866**	0.422	0.313
**MF**	**Prote**	-.915**	-.954**	0.023	-0.471	-0.552	-.581*	-.630*	-0.545	-0.326
	**Actin**	-.892**	-.945**	0.004	-0.437	-0.541	-0.566	-.637*	-0.561	-0.295
	**Amoun**	.894**	.874**	0.015	0.504	0.528	0.57	.686*	0.485	0.417
	**Abund**	.886**	.897**	0.021	0.508	0.562	.583*	.700*	0.514	0.366
	**Shannon**	-0.211	0.381	0.052	-0.334	0.076	-0.447	-.730**	0.371	-.919**
	**Richness**	-.820**	-.775**	0.331	-0.006	0.018	0.393	-0.031	-0.516	0.122
	**ShannonG**	.763**	.878**	.592*	-0.078	-0.13	-0.564	-0.173	.663*	-0.365
**SF**	**Prote**	-.623*	-.711**	0.302	0.129	-0.234	0.376	0.361	-0.501	.674*
	**Actin**	-.685*	-.709**	0.322	0.093	-0.254	0.323	0.284	-0.466	.614*
	**Amoun**	.734**	.953**	-0.071	-0.205	-0.017	-0.548	-0.226	.671*	-0.514
	**Abund**	.749**	.950**	-0.096	-0.153	0.064	-0.525	-0.223	.646*	-0.533
	**Shannon**	-.594*	-.831**	-0.044	-0.118	-0.036	-0.138	-.751**	-0.005	-.717**
	**Richness**	-.650*	-0.158	0.174	-0.052	0.092	0.018	0.159	-0.086	0.393
	**ShannonG**	0.591*	-.601*	.603*	0.239	0.513	.583*	-0.134	-0.053	0.034
**MPF**	**Prote**	-.727**	0.321	0.244	-0.243	-0.119	-0.166	0.078	0.004	0.237
	**Actin**	-.707*	0.357	0.247	-0.238	-0.113	-0.157	0.103	0.028	0.245
	**Amoun**	.669*	-0.43	-0.358	0.27	0.144	0.176	-0.105	-0.008	-0.178
	**Abund**	.635*	-0.483	-0.291	0.276	0.133	0.158	-0.15	-0.05	-0.19

Notes: Pearson correlation was used to examine the correlations between soil properties and microbial properties. *, $P < 0.05$; **, $P < 0.01$; R, Pearson's correlation coefficient. Abbreviations: ST, soil temperature; SM, soil moisture; CEC, cation exchange capacity; TN, total nitrogen; TP, total

phosphorus; SOM, soil organic matter; Richness and Shannon, diversity index of microbial community; ShannonG, diversity index of N mineralization genes; Amoun, amount of N mineralization genes; Abund, abundance of N mineralization genes; Prote, Proteobacteria, Actin, Actinobacteria.
